# Cardiomyocyte Rac1 signaling in hypertrophy, arrhythmia, and cardiac stress adaptation

**DOI:** 10.1016/j.jmccpl.2025.100826

**Published:** 2025-11-14

**Authors:** James P. Teuber, Rachel E. Scissors, Matthew J. Brody

**Affiliations:** a Department of Pharmacology, University of Michigan, Ann Arbor, MI, USA; b Department of Internal Medicine, University of Michigan, Ann Arbor, MI, USA

**Keywords:** Rac1, PAK1, NOX2, ASK1, Cardiac hypertrophy, Cardiomyocytes, Protein kinase A, Arrhythmia, Palmitoylation, Heart failure

## Abstract

Cardiovascular disease remains the leading cause of mortality globally and is often marked by pathologic cardiac remodeling including hypertrophy and fibrosis that promote the progression to heart failure. Ras-related C3 botulinum toxin substrate 1 (Rac1) is a Rho family small GTPase that acts as a molecular switch to regulate signaling pathways that contribute to cardiac development, hypertrophy, arrhythmia, and stress adaptation. Active Rac1 promotes cardiomyocyte hypertrophy *in vitro* and *in vivo* whereas genetic ablation or expression of inactive Rac1 protects against cardiomyocyte hypertrophy. Rac1 activates mitogen-activated protein kinase (MAPK) cascades and its canonical effector, p21-activated kinase 1 (PAK1), to promote hypertrophic gene expression. Additionally, Rac1 is a requisite accessory subunit required to activate the reactive oxygen species (ROS)-generating NADPH oxidase-2 (NOX2) enzyme complex that in turn induces hypertrophic redox signaling and oxidative damage. Cardiomyocyte Rac1 activity plays an indispensable function in cardiac adaption to elevated sympathetic activity. Rac1 cysteine palmitoylation cycling is required to attenuate hyperactive protein kinase A (PKA) signaling in response to acute adrenergic stimulation and in several models of chronic hypertrophic stress. Moreover, Rac1 and its effectors have important roles in cardiomyocyte electrophysiology and arrhythmogenesis and therapeutic approaches directly targeting Rac1, NOX2, PAK1, or apoptosis signal-regulating kinase 1 (ASK1) have shown promise in preclinical models of cardiac disease. Here, we review what is known about Rac1 signaling in cardiomyocytes, discuss how these signaling pathways can potentially be targeted for the treatment and prevention of cardiac disease, and propose areas of Rac1 signaling that warrant further exploration.

## Graphical abstract

**Figure:**
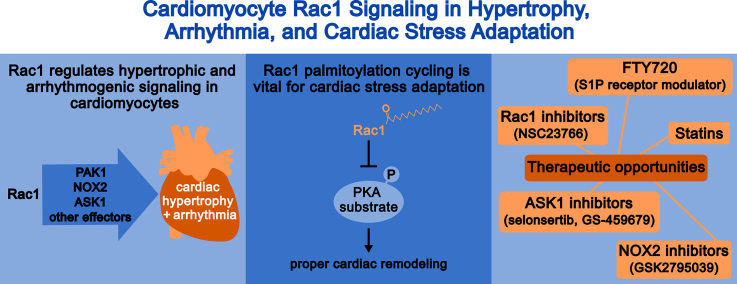

## 1 Introduction

Cardiovascular disease, including heart failure, is the leading cause of morbidity and mortality in the United States [1,2]. Pathologic cardiac remodeling, often associated with cardiac hypertrophy and cardiac arrhythmias, promotes the progression of heart failure [3]. While decades of preclinical research have aided our understanding of the intracellular signaling pathways that regulate cardiac remodeling, there remains an unmet need to further elucidate these pathways and identify novel therapeutic targets for the treatment and prevention of these devastating diseases. Ras-related C3 botulinum toxin substrate 1 (Rac1) activity is elevated in failing human hearts [4,5] and in many animal models of pathologic cardiac remodeling [[6], [7], [8]]. In this review, we will highlight what is known about Rac1 signaling in cardiomyocytes and cardiac pathologies and discuss future directions and therapeutic opportunities related to Rac1 signaling.

Rac1 is a member of the Rho family of small GTPases that serve as molecular switches regulated by their binding to guanine nucleotides [[9], [10], [11]]. Like all large and small G proteins, Rac1 cycles between inactive GDP-bound and active GTP-bound states and this cycling is regulated by guanine exchange factors (GEFs) and GTPase-activating proteins (GAPs) which load and hydrolyze GTP, respectively [9,12]. Rho guanine dissociation inhibitors (GDIs) further regulate the activity and trafficking of Rac1 by functioning as a cytosolic chaperone to shuttle Rac1 throughout the cell while shielding its hydrophobic isoprenyl group from the aqueous cytoplasm [12]. Rac1 primarily exists as the 192 amino-acid isoform, Rac1a, although it can be alternatively spliced to include a 19 amino acid loop resulting in the 211 amino acid Rac1b isoform that has reduced membrane localization and impaired prenylation [13]. In the heart, Rac1a is the best studied isoform and will be referred to as Rac1 throughout this review.

In addition to guanine nucleotide cycling, Rac1 is regulated by several post-translational modifications [14]. During protein maturation, Rac1 is geranylgeranylated (20‑carbon isoprenylation) at the endoplasmic reticulum membrane at cysteine-189 [[15], [16], [17]] that, along with its C-terminal polybasic domain, enhances Rac1 localization to intracellular membranes [18]. Following isoprenylation, Rac1 is further modified by cleavage of the three most C-terminal amino acids (LLL) by Ras converting enzyme (RCE) and subsequent carboxymethylation of the prenylated cysteine-189 by isoprenylcysteine carboxyl methyltransferase (ICMT) [19,20]. Additionally, Rac1 undergoes palmitoylation/depalmitoylation cycling at cysteine-178 that allows for the dynamic addition and removal of a second lipid anchor on its C-terminus that modulates insertion into membrane microdomains [[21], [22], [23], [24]]. Rac1 is also reported to be phosphorylated at serine-71 by Akt to negatively regulate its activity [25,26], however the functional consequences of this modification in the heart remain unknown.

Rac1 has been implicated in the molecular etiology of a plethora of disease processes including cancer, neurodevelopmental disorders, inflammatory conditions, and renal disease [11]. On a molecular level, Rac1 is perhaps best characterized for its ability to induce lamellipodia that drives cytoskeletal remodeling to facilitate cell migration [9]. However, cardiomyocytes are terminally differentiated cells with a complex intracellular membrane cytoarchitecture, myofilament- and mitochondria-dominated cytoplasmic space, and limited dynamics of the cortical actin cytoskeleton that have made ascertaining the precise molecular functions of Rac1 in cardiac myocytes particularly elusive. Here, we will review what is known about Rac1 signaling in cardiac stress adaptation and disease pathogenesis, highlight the functions of the primary cardiomyocyte Rac1 effectors, NADPH oxidase 2 (NOX2), p21-activated kinase 1 (PAK1), and apoptosis signal-regulated kinase 1 (ASK1), and discuss relevant therapeutic opportunities.

## 2 Rac1 signaling in cardiac hypertrophy

**Fig. 1:**
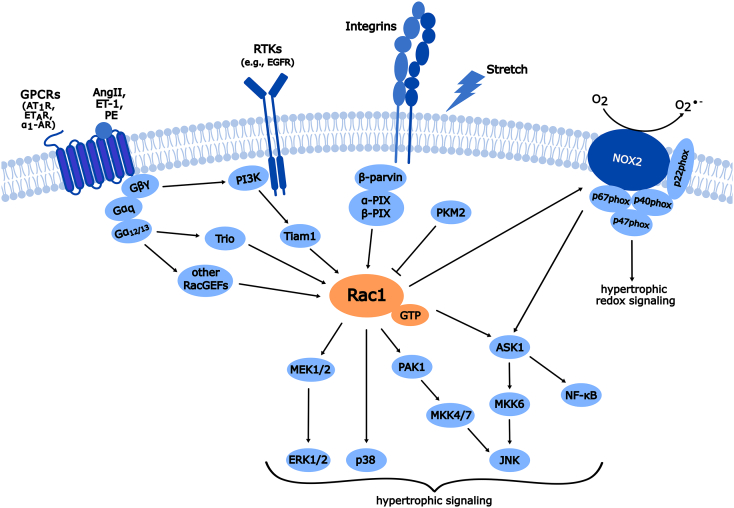
**Rac1 signaling in cardiomyocyte hypertrophy.** Rac1 is activated downstream of several stimuli including G protein coupled receptors (GPCRs), receptor tyrosine kinases (RTKs), integrins, and mechanical stretch. These signals are transduced through activation of Rac GEFs including Trio, Tiam1, α-pix, and β-pix among others. This results in the activation and GTP loading of Rac1 leading to activation of MAPK, PAK1, and NOX2 signaling. Rac1 activation is inhibited by pyruvate kinase M2 (PKM2)-mediated phosphorylation. Rac1-dependent activation of apoptosis signaling kinase 1 (ASK1) results in activation of nuclear factor kappa B (NF-κB) and induction of MAPK cascades including MEK/ERK, p38, and JNK signaling, which modulate hypertrophic remodeling. Rac1-dependent activation of PAK1 in cardiomyocytes leads to enhanced JNK signaling and antagonizes cardiac hypertrophy. Rac1 activates NADPH oxidase-2 (NOX2) along with auxiliary subunits (p67^phox^, p47^phox^, p40^phox^) which results in superoxide production that promotes hypertrophic redox signaling.

**Fig. 2:**
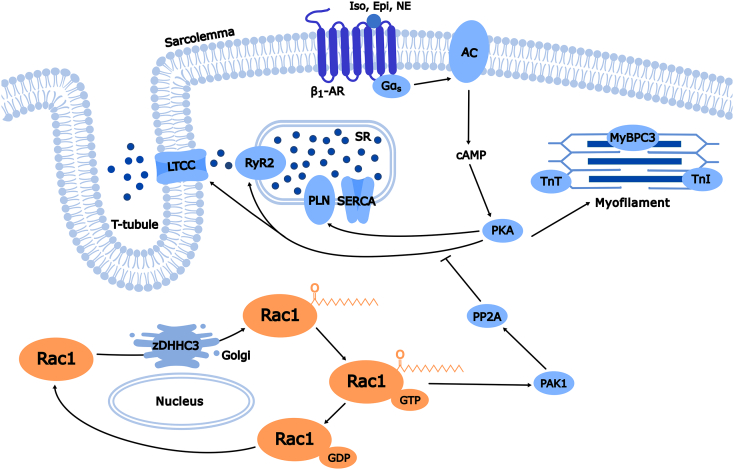
**Palmitoylation of Rac1 at cysteine-178 regulates cardiomyocyte adrenergic signaling.** Activation of the β1-adrenergic receptor (β1-AR) with isoproterenol or (nor)epinephrine leads to cAMP production and PKA activation, resulting in phosphorylation of calcium handling (*e.g.*, LTCC, RyR2, PLN) and contractile proteins (*e.g.*, TnT, TnI, MyBPC3). Palmitoylation cycling of Rac1 at cysteine-178 is critical for restraining hyperactive PKA signaling in the stressed heart, potential through regulation of localized activation of PAK1 and PP2A phosphatase activity, which negatively regulates PKA-regulated phospho-sites on substrate proteins. Loss of Rac1 palmitoylation cycling at cysteine-178 leads to PKA substrate hyperphosphorylation that accelerates cardiac maladaptation.

Rac1 signaling in cardiac myocytes has been best studied as a mediator of pathologic hypertrophic signaling. Rac1 is activated in response to a myriad of hypertrophic stimuli, including but not limited to angiotensin II (AngII), phenylephrine (PE) [27,28], endothelin-1 (ET-1) [28,29], stretch [30,31], pressure overload [6] and in multiple animal models of hypertension-induced heart disease [32,33]. Therefore, it is perhaps unsurprising that Rac1 plays critical roles in cardiac remodeling in response to hypertrophic stress (Fig. 1, Fig. 2).

### 2.1 Studies in isolated cardiomyocytes

Initial studies identified that overexpression of constitutively-active Rac1^G12V^ in cardiomyocytes induced hypertrophy of neonatal rat cardiomyocytes (NRCMs) whereas expression of constitutively inactive Rac1^T17N^ reduced PE-stimulated increases in protein synthesis, an indicator of cardiomyocyte hypertrophy [34]. Expression of Rac1^T17N^ also blunted ET-1-induced elevation of β_1_-integrin expression, reactive oxygen species (ROS) production, and activation of epidermal growth factor receptor (EGFR)/mitogen-activated protein kinase kinase 1 (MEK) and downstream ERK1/2 signaling [28,29]. Therefore, it was proposed that Rac1-dependent superoxide production may regulate EGFR transactivation following endothelin receptor (ET_A_R) activation to promote cardiomyocyte hypertrophy [29]. Hyperactive Rac1^G12V^ was found to activate ASK1 and nuclear factor kappa B (NF-κB) and blockade of ASK1/NFκB signaling mitigated Rac1^G12V^-induced cardiomyocyte hypertrophy [35] whereas overexpression of dominant-negative Rac1^T17N^ attenuated PE-evoked ASK1 activation and cardiomyocyte hypertrophy [35]. The pro-hypertrophic effects of Rac1^G12V^ expression were also prevented by *N*-acetylcysteine treatment, suggesting ROS is involved in Rac1-regulated activation of ASK1/NFκB signaling in cardiomyocytes [35]. α_1_-adrenergic receptor (α_1_-AR) activation in NRCMs by PE also elicits Rac1 activation and hypertrophy that is impaired by inhibition of Gβγ, phosphoinositide 3-kinase (PI3K), or the RacGEF T-cell lymphoma invasion and metastasis-inducing protein1 (Tiam1), suggesting Gβγ-mediated activation of PI3K and Tiam1 provoke Rac1-dependent cardiomyocyte hypertrophy [27]. Therefore, it is plausible that Gβγ may mediate Rac1 activation following the activation of other G protein-coupled receptors (GPCRs) through PI3K/Tiam1 (Fig. 1).

Rac1 was also shown to be activated in response to stretch in NRCMs, which preferentially shifted localization of Rac1 from caveolin-rich lipid rafts to the cytoplasm and was blocked by methyl-β-cyclodextrin (MBCD) treatment [30], suggesting integrity of lipid rafts is required for stretch-dependent activation of Rac1 in cardiomyocytes. Stretch-induced Rac1 activation and cardiomyocyte hypertrophy were also shown to be blocked by expression of dominant-negative protein kinase C (PKC) [31]. Furthermore, Rac1^G12V^ was shown to activate p38 MAPK while Rac1^T17N^ blocked stretch-induced p38 MAPK activation and protein synthesis in NRCMs [36]. Rac1 has also been reported to be activated downstream of integrins and knockdown of β-parvin in NRCMs reduced basal Rac1 GTP-loading and hypertrophic growth over three days in culture, similar to treatment with the Rac1 inhibitor EHT1864, suggesting a role for β-parvin in mediating Rac1-dependent hypertrophy downstream of integrin activation at focal adhesions/costameres [37]. Taken together, studies using genetic manipulation of Rac1 expression and activity in primary cardiomyocytes have established important roles for Rac1-regulated MAPK signaling and ROS production in cardiomyocyte hypertrophy (Fig. 1).

### 2.2 Studies in animal models of pathologic cardiac hypertrophy

The first evidence of Rac1 involvement in cardiac hypertrophy *in vivo* resulted from the generation of transgenic mice expressing Rac1^G12V^ (also called Rac1^ET^ mice), which were found to exhibit lethal dilated cardiomyopathy, although a fraction of mice displayed transient perinatal hypertrophy that normalized with age [38]. A second model of hyperactive Rac1 signaling was developed by expressing a constitutively active corn Rac transgene, ZmRacD, in mice, which also resulted in cardiac hypertrophy and reduced systolic function with age [39].

Because global deletion of Rac1 in mice is embryonically lethal [40], cardiomyocyte-specific Rac1 knockout mice were generated to assess the necessity of cardiomyocyte Rac1 signaling in cardiac hypertrophy. Cardiomyocyte-specific deletion of Rac1 protected mice from AngII-induced cardiac hypertrophy, which was associated with reduced NOX2 activity and superoxide production as well as reduced ASK1/NFκB activation [41]. Heterozygous loss of Rac1 in cardiomyocytes, or treatment with the Rac1 inhibitor NSC23766, limited cardiac hypertrophy and improved systolic function following transverse aortic constriction (TAC)-induced pressure overload in association with blunted mineralocorticoid receptor activation and NOX activity [6].

Rac1 deletion in cardiomyocytes was also found to be protective in a model of streptozotocin-induced diabetic cardiomyopathy [42,43]. Cardiomyocyte-specific Rac1 knockout mice were protected from streptozotocin-induced cardiac hypertrophy and fibrosis and defects in cardiac contraction and relaxation while administration of the Rac1 inhibitor, NSC23766, reduced superoxide production and apoptosis observed in diabetic *db/db* mice, however this did not affect cardiac hemodynamics [42]. Expression of dominant-negative Rac1^T17N^ or Rac1 deletion in cardiomyocytes attenuated streptozotocin-stimulated increases in NOX activity, endoplasmic reticulum (ER) stress, and apoptosis [42].

Recent work from our group elucidated a role for palmitoylation cycling of Rac1 at cysteine-178 as a crucial regulatory mechanism for cardiac stress adaptation. Cardiomyocyte-specific overexpression of the Golgi-localized palmitoylating S-acyltransferase enzyme, zDHHC3, resulted in severe dilated cardiomyopathy which coincided with increased palmitoylated and active Rac1 levels [22]. To directly assess the functions of Rac1 palmitoylation *in vivo*, we generated cardiomyocyte-specific Rac1 conditional knock-in (Rac1^cKI^) mice expressing palmitoylation-resistant Rac1^C178S^ and found that palmitoylation at cysteine-178 is dispensable for cardiac homeostasis as Rac1^cKI^ mice do not exhibit changes in cardiac structure or function with age [24]. However, in response to multiple models of chronic hypertrophic stress including AngII infusion, TAC-induced pressure overload, or transgenic overexpression of the AngII type 1 receptor (AT1R) in cardiomyocytes, Rac1^cKI^ mice develop exacerbated cardiac hypertrophy and display accelerated functional decompensation [24]. This was associated with hyperphosphorylation of protein kinase A (PKA) substrates in Rac1^cKI^ hearts following chronic hypertrophic stress or acute β-adrenergic stimulation without changes in PKA enzymatic activity, suggesting loss of Rac1 palmitoylation may impair trafficking and/or activation of protein phosphatases that negatively regulate PKA phospho-sites in cardiomyocytes [24]. Exactly how Rac1, and palmitoylation of Rac1 at cysteine-178, regulate remodeling of the PKA-dependent phosphoproteome remains incompletely understood and warrants further investigation.

Importantly, sex differences in Rac1 signaling have been incompletely explored. While most experiments using pathologic cardiac hypertrophy models discussed above have been performed in animals of both sexes and did not report sex-dependent differences, there is limited evidence indicating potential sex differences in cardiomyocyte Rac1 signaling. Deletion of the β-parvin-interacting protein, syndecan-4, resulted in shorter cardiomyocytes in females that was associated with reduced Rac1 membrane levels and increased Rac1 activity whereas Rac1 activity was reduced and membrane levels increased in left ventricles of male syndecan-4-deleted mice [44]. Although the precise mechanisms remain unclear, these data suggest that Rac1 activation mediated through the β-parvin/β-PIX complex may be regulated differently between sexes. Roles for estrogen in regulation cardiac Rac1 activity have been reported. Ovariectomized female transgenic mice with global overexpression of coupling factor 6 (CF6) have increased Rac1 activity that is normalized with estrogen supplementation [45]. In addition, lipopolysaccharide (LPS)-stimulated Rac1 activation in the heart is greater in male compared to female mice and LPS-mediated induction of Rac1 mRNA and total protein levels as well as NOX2 activity and ROS production are greater in male mice but normalized to levels in females by administration of estrogen [46]. These data suggest Rac1 activity in the heart is negatively regulated by estrogen although rigorous studies simultaneously evaluating male and female cardiomyocyte-specific Rac1 gene-deleted mice subjected to hypertrophic stress models would aid in understanding the direct mechanisms by which cardiomyocyte Rac1 is regulated in a sex-dependent manner.

### 2.3 Rac1 in physiologic cardiac hypertrophy

As Rac1 is primarily associated with pathologic hypertrophic signaling, there is comparatively much less known about the function of Rac1 in physiologic cardiac growth. There is limited evidence describing reduced Rac1 protein levels in the rat heart following chronic exercise [47], however it is highly possible that this effect is not driven by cardiomyocyte Rac1, as Rac1 is appreciably expressed in several other cardiac cell types. Insulin and insulin-like growth factor-1 (IGF-1)-are common drivers of physiologic cardiac growth [3]. In skeletal muscle, Rac1 mediates insulin-induced GLUT4 translocation to the sarcolemma [48] and loss of Rac1 in skeletal muscle reduces insulin- or exercise-induced glucose uptake *in vivo* [49,50]. Furthermore, endurance training was shown to increase Rac1 protein levels in skeletal muscle and loss of Rac1 in skeletal muscle reduced exercise-induced skeletal muscle hypertrophy and protein synthesis [51]. Whether Rac1 mediates similar effects in response to insulin/IGF-1 stimulation and exercise in the cardiac myocyte remains unexplored. Direct assessment of the effect of cardiomyocyte-specific Rac1 deletion on cardiac structure, function, and signaling in response to established physiologic hypertrophy models such as chronic swimming and running would provide important context to the rationale of targeting Rac1 for heart disease.

### 2.4 Rac1-interacting proteins in cardiac hypertrophy

Several studies have identified roles of proteins that interact with Rac1 in the development of cardiac hypertrophy. Global deletion of dedicator of cytokinesis 10 (DOCK10), a Rac GEF, resulted in more severe cardiac hypertrophy following AngII infusion and a greater reduction in p38 and JNK activation following acute PE treatment, however no direct effects on Rac1 activation were demonstrated [52]. Additionally, it was shown that the GEF, differentially expressed in FDCP6 homolog (DEF6), exacerbates cardiac hypertrophy in response to pressure overload [53]. DEF6 and Rac1 were shown to physically interact and NSC23766 treatment blunted MEK/ERK signaling and NRCM hypertrophy caused by DEF6 overexpression [53]. However, whether DEF6 acts directly as a Rac1 GEF or activates Rac1 indirectly remains undetermined.

Proteomic studies identified six-transmembrane epithelial antigen of prostate 3 (STEAP3) as an interactor of Rac1 that was confirmed in NRCMs [54]. STEAP3 was downregulated in response to hypertrophic stimuli and deletion of STEAP3 exacerbated cardiac hypertrophy in response to TAC whereas STEAP3 overexpression suppressed MEK/ERK signaling and cardiomyocyte hypertrophy that was circumvented by overexpression of constitutively-active Rac1^G12V^ [54], suggesting STEAP3 may antagonize pathologic cardiomyocyte hypertrophy through repression of Rac1. However, the exact mechanisms by which STEAP3 influences Rac1 signaling and cardiac remodeling remain unclear.

Lysosomal-associated protein transmembrane 5 (LAPTM5) was shown to interact with Rac1 in HEK cells and its overexpression reduced MEK/ERK activation and hypertrophy in NRCMs in response to AngII that was restored by overexpression of hyperactive Rac1^G12V^ [55], suggesting LAPTM5 binding may sequester Rac1 to prevent its activation of hypertrophic MEK/ERK signaling. Indeed, deletion of LAPTM5 was found to exacerbate pressure overload-induced cardiac hypertrophy and MAPK/ERK signaling while overexpression of LAPTM5 in the heart reduced cardiac hypertrophy and MAPK/ERK activation in response to pressure overload [55].

Pyruvate kinase M2 (PKM2) has been proposed to negatively regulate Rac1 signaling activity in cardiac remodeling through direct phosphorylation. PKM2 was found to interact with Rac1 in cardiomyocytes and PKM2 deficiency worsened TAC-induced cardiac hypertrophy [56]. Knockdown of PKM2 reduced Rac1 serine-71 phosphorylation and increased Rac1 protein stability and activation of JNK and p38 MAPK cascades, but not ERK1/2 [56]. Treatment with the Rac1 inhibitor NSC23766 ameliorated more severe hypertrophy and left ventricular dysfunction observed in TAC-operated PKM2-knockout mice, suggesting PKM2-mediated phosphorylation and repression of Rac1 may limit pathologic cardiac remodeling in response to pressure overload [56]. Thus, there are several proteins that modulate Rac1 in cardiomyocytes that impact cardiac remodeling and stress adaptation.

### 2.5 Statins in cardiac hypertrophy

Statins were developed as a cholesterol-lowering therapy and have been successful at reducing the severity of heart disease in humans. Acting primarily as HMG-CoA reductase inhibitors, it has become clear that stains have beneficial effects in addition to lowering systemic cholesterol levels [57]. Specifically, statins inhibit the conversion of HMG-CoA to mevalonate which is a precursor to isoprenyl groups including geranylgeranyl-pyrophosphate, an intermediate required for the isoprenylation of Rac1 that is necessary for its processing, intracellular trafficking, and function [58]. Several lines of evidence demonstrate an association of statin-mediated cardioprotection with impaired Rac1 activity or abundance in the heart. Indeed, right atrial tissue from humans treated with atorvastatin or pravastatin displayed reduced active and total Rac1 levels along with decreased NOX activity [5]. Atorvastatin and pitavastatin were shown to reduce membrane-associated Rac1 and active Rac1 levels in response to AngII infusion in rats [59] and treatment with simvastatin [60] or atorvastatin [61] reduced the degree of cardiac hypertrophy in mice subjected to left ventricular pressure overload by TAC.

Expression of dominant negative Rac1 or treatment with atorvastatin or a geranylgeranyl-transferase inhibitor, GGTI-286, prevented AngII-induced Rac1 activation and cardiomyocyte hypertrophy [61]. Treatment with rosuvastatin was found to attenuate pressure overload-induced increases in Rac1 activity, NOX2 activity, and Rac1 interaction with RhoGDIα [62]. Furthermore, knockdown of RhoGDIα in H9C2 cardiomyocyte-like cells inhibited AngII-induced superoxide production and protein synthesis, suggesting association of Rac1 with RhoGDIα may be critical for AngII-regulated NOX2 activity and hypertrophy [62].

In a rabbit model of *Myh7*-Q403-driven cardiac hypertrophy, treatment with simvastatin reduced ERK phosphorylation and did not affect Rac1 GTP-loading, however it did appear to mildly reduce Rac1 membrane localization [63]. Treatment of *Myh7*-Q403 rabbits with atorvastatin reduced cardiac hypertrophy and fibrosis, however the effect of atorvastatin was reported to not affect Rac1 activity or membrane localization in these animals, although these data were not shown [64]. In contrast, treatment with simvastatin ameliorated Rac1 GTP-binding, superoxide production, and cardiac hypertrophy in response to AngII in isolated rat cardiomyocytes and rat hearts [60] and treatment with cerivastatin or expression of GDP-locked Rac1^T17N^ inhibited epinephrine-evoked Rac1 activation, cardiomyocyte apoptosis, and JNK signaling [65]. Similarly, simvastatin administration reduced AngII-induced Rac1 membrane localization and activation in H9c2 cells and atorvastatin-treated rats had reduced cardiac Rac1 activity and atrial natriuretic peptide (ANP) expression [66]. Thus, while statins reduce Rac1 activation and membrane localization as a consequence of impaired isoprenoid biosynthesis that is associated with improved cardiac pathology, the effects of statins on Rac1 signaling may be dose and context-dependent.

### 2.6 NOX2 in cardiac hypertrophy

One unique aspect of Rac1 signaling compared to other small GTPases is the ability to stimulate ROS production through activation of NOX2. Among NOX isoforms, NOX1 and NOX2 are the only two that require Rac1 activation to be catalytically active [67,68]. In cardiomyocytes, NOX2 and NOX4 are the predominant isoforms and thus Rac1-dependent ROS generation in cardiomyocytes is largely mediated by NOX2-regulated superoxide production at the sarcolemma. The NOX2 catalytic subunit, gp91^phox^, exists as a membrane-embedded heterodimer in complex with p22^phox^ in the absence of stimulation and forms a multimeric enzymatic complex following Rac1-dependent recruitment of p67^phox^, p47^phox^, and p40^phox^ to the plasma membrane [68]. Rac1-mediated NOX2 activity is proposed to regulate hypertrophic signaling. Indeed, NOX activity and p47^phox^ membrane localization are increased in human cardiomyopathy [5] and NRCMs expressing dominant-negative Rac1^T17N^ or with siRNA-mediated knockdown of Nox2 are protected from AngII-induced ROS generation and cardiomyocyte hypertrophy [69], suggesting Rac1/NOX2 signaling is critical for AngII-induced cardiac hypertrophy.

*In vivo*, deletion of the NOX2 auxiliary subunit, p47^phox^ impaired AngII-induced cardiac hypertrophy and activation of ERK1/2, p38, and JNK MAPKs and ASK1, suggesting NOX2-mediated redox regulation of these signaling kinases [70]. Indeed, ASK1 has been shown to be activated in response to ROS and in turn lead to p38 MAPK activation [71] and NOX2-generated ROS initiates prohypertrophic redox signaling including oxidation-dependent activation of CaMKII [72,73]. *Nox2* (*Cybb*/gp91^phox^) gene-deleted mice are also protected from AngII-induced cardiac hypertrophy and fibrosis [74]. While initial studies suggested that *Nox2*^−/−^ mice were not protected from pressure overload-induced cardiac hypertrophy [75,76], more recent studies have reported protection from pressure overload-induced cardiac hypertrophy and fibrosis and improved cardiac hemodynamics compared to wildtype mice, which was associated with reduced activation of ERK1/2, p38, and JNK MAPK signaling cascades [77]. *Nox2*-null mice are also protected from high fat diet-induced cardiac hypertrophy and ROS production [78] and cardiac hypertrophy, fibrosis, and systolic functional deficits following myocardial infarction [79]. Importantly, this widely used *Nox2* knockout model was later shown to express a truncated, inactive Nox2 protein [80], although several laboratories have demonstrated defects in NOX2-regulated superoxide production [81]. In contrast, NOX2 transgenic mice develop more severe cardiac hypertrophy following AngII infusion or TAC [82]. More recently, cardiomyocyte-specific NOX2 transgenic mice, but not endothelial cell-specific NOX2 transgenic mice, were shown to have worsened survival, fibrosis, and hypertrophy following myocardial infarction, suggesting cardiomyocyte NOX2 mediates most of its pathogenic effects on the heart [83].

### 2.7 PAK1/2 in cardiac hypertrophy

p21-activated kinases 1 and 2 (PAK1/2) have important roles in opposing maladaptive cardiac hypertrophy as reviewed elsewhere [84,85]. Briefly, it is known that PAK1 can be activated both by Rac1 and another Rho family small GTPase, Cdc42 [84]. Cardiomyocyte-specific deletion of *Pak1* does not affect basal cardiac structure or function but results in exacerbated cardiac hypertrophy following TAC or AngII infusion [86]. Loss of PAK1 impaired cardiac MKK4/7 and JNK activation in response to TAC without impaired activation of other MAPKs, suggesting a PAK1/MKK4/7/JNK pathway represses hypertrophy [86]. Additionally, PAK1 knockdown in NRCMs exacerbated PE-stimulated hypertrophy and nuclear factor of activated T-cell (NFAT) transcriptional activity [86], suggesting an antihypertrophic role for PAK1 signaling potentially through JNK-mediated repression of calcineurin in cardiomyocytes [87]. PAK2 is also activated in response to TAC and cardiomyocyte-specific deletion of *Pak2* results in worsened systolic function and more severe hypertrophy following pressure overload that was associated with impaired ER stress [88]. In contrast to pathological hypertrophy models, global *Pak1* deletion was shown to reduce endurance capacity and physiologic cardiac hypertrophy following exercise training that was associated with blunted cardiac contractility, myofilament calcium sensitivity, and myofilament protein phosphorylation [89].

FTY720 (fingolimod) is an FDA-approved drug for the treatment of multiple sclerosis [90]. Administration of FTY720, a sphingosine-1-phosphate (S1P) receptor modulator, protects against TAC-induced cardiac hypertrophy and preserves systolic function in wildtype mice, but not in mice with cardiomyocyte-specific loss of PAK1, suggesting FTY720 is cardioprotective through a PAK1-dependent pathway [86]. FTY720 does activate PAK1 in NRCMs and adult mouse hearts [86] and blocks NFAT nuclear translocation and ANP expression in response to PE in control NRCMs but not those with shRNA-mediated knockdown of PAK1 [91]. FTY720 is reported to exert its effects as the modified FTY720-phosphate and act through the Gα_i_-coupled S1P receptor (S1PR1), as treatment with sphingosine kinase (SPHK) inhibitors or pertussis toxin, or competition with sphingosine-1-phosphate, block FTY720-induced PAK1 activation in NRCMs [91]. Moreover, treatment with pertussis toxin blocks the antihypertrophic effects of FTY720 in response to TAC *in vivo* [91]. Additionally, FTY720 administration reduced left atrial size and improved diastolic function in a mouse model of hypertrophic cardiomyopathy caused by the tropomyosin E180G mutation [92] and reduced infarct size and cardiac hypertrophy, fibrosis, and systolic dysfunction in a porcine model of ischemia-reperfusion (I/R) injury [93].

### 2.8 ASK1 in cardiac hypertrophy

In addition to well-characterized functions of PAK1 and NOX2 in the heart, there is a growing body of evidence demonstrating roles for ASK1 in cardiac remodeling. ASK1 is activated in response to hypertrophic stimuli including AngII [71], ET-1 [94], and PE [94], as well as ROS [71]. ASK1 activation was prevented by deletion of the NOX2 auxiliary subunit, p47^phox^, further supporting that ASK1 is activated *via* a redox-sensitive mechanism [70] and suggesting Rac1-dependent NOX2-mediated ROS production promotes pathologic redox signaling in part through activation of ASK1.

Global loss of ASK1 in *Map3k5*^−/−^ mice resulted in no basal cardiac phenotype but improved function, reduced ventricular dilation, and reduced cardiomyocyte apoptosis following myocardial infarction or pressure overload [95] and reduced hypertrophy following AngII infusion [96]. Furthermore, cardiomyocyte-specific deletion of ASK1 is protective against pressure overload-induced cardiac hypertrophy and dysfunction [97]. Cardiac-specific ASK1 overexpression did not induce cardiac hypertrophy, however ASK1 transgenic mice displayed exacerbated dysfunction and increased infarct area size and cardiomyocyte apoptosis following myocardial infarction [98]. Furthermore, cardiac ASK1 overexpression induced exacerbated cardiac hypertrophy, fibrosis, dysfunction, and apoptosis following long-term pressure overload that was associated with enhanced ASK1-mediated phosphorylation of MKK6 and JNK activation [98], suggesting a role for ASK1/MKK6/JNK signaling in cardiac maladaptation to pathologic stimuli. ASK1 also plays roles in negatively regulating physiologic cardiac remodeling as *Map3k5*^−/−^ mice developed more pronounced cardiac hypertrophy in response to swimming that was associated with increased cardiac Akt signaling and impaired p38 MAPK activation [99].

Several layers of regulation exist to modulate ASK1 activity within the cardiac myocyte. Tumor necrosis receptor-associated factor 7 (TRAF7), an E3 ubiquitin ligase, facilitates K63-linked ubiquitination of ASK1 at Lys-1064, which promotes ASK1 activation in response to hypertrophic stimuli and enhances pathologic remodeling [97]. Binding of lipopolysaccharide-induced tumor necrosis factor-alpha factor (LITAF) to the ASK1 N-terminus prevents ASK1 activation and reduces hypertrophy in response to PE or pressure overload [100]. Direct binding of dual specificity phosphatase 9 (DUSP9) or *N*-acetylgalactosaminyltransferase 4 (GALNT4) to ASK1 also reduce its activation, which protects against pathologic cardiac hypertrophy *in vitro* and *in vivo* [101,102]. The identification of additional molecular regulators of ASK1 activation will provide new tractable therapeutic targets for the amelioration of cardiac pathology.

Therapeutic strategies directly inhibiting ASK1 activation have shown promise in preclinical models of cardiac hypertrophy and failure. Indeed, treatment with the ASK1 inhibitor selonsertib ameliorated AngII-induced cardiac hypertrophy and fibrosis in mice [71] and treatment with GS-459679, another ASK1 inhibitor, reduced infarct size and improved systolic function following I/R injury [103]. Thus, blockade of the Rac1-NOX2-ASK1 signaling cascade *via* direct inhibition of ASK1 or its activators may be a viable therapeutic strategy for limiting the progression of cardiac disease.

## 3 Rac1 signaling in cardiac arrhythmia and electrophysiology

### 3.1 Rac1 in cardiac arrhythmia

Early discovery that RhoGDIα overexpression in cardiomyocytes decreased Rho GTPase activity and induced arrhythmias and atrioventricular block [104] led to the study of Rac1 in cardiac arrhythmias. Cardiomyocyte-specific Rac1 knockout mouse embryos show defects in myocardial development such as ventricular noncompaction that acts as a substrate for arrhythmias [105,106]. Human left atrial samples from patients with atrial fibrillation (AF) show upregulated Rac1 GTPase activity compared to patients with sinus rhythm, and Rac1^ET^ mice develop AF by 16 months of age [107]. Cardiac structural remodeling such as fibrosis often acts as a substrate for AF and other conduction abnormalities, with higher levels of interstitial fibrosis found in both AF patient and Rac1^ET^ mouse atrial tissue [[107], [108], [109]].

Rac1-mediated regulation of multiple proteins, including signal transducer and activator of transcription 3 (STAT3) and connective tissue growth factor (CTGF), contributes to fibrotic remodeling that supports the development of AF. The atria of patients with AF as well as atria from a porcine model of pacing-induced sustained AF have increased tissue levels of AngII and active STAT3 [[110], [111], [112]]. STAT3 activates proinflammatory pathways and alters protein expression that may contribute to atrial structural remodeling [110]. In isolated atrial myocytes, STAT3 is activated by AngII treatment or transfection with constitutively active Rac1 [112]. AngII-induced STAT3 activation in atrial myocytes is Rac1-dependent, as expression of dominant-negative Rac1 abolishes the increase in phosphorylated STAT3 [112]. In rats, AngII infusion similarly increased levels of active STAT3 and fibrosis in the atria, both of which were blocked by treatment with simvastatin [112]. STAT3 nuclear translocation and DNA binding increased in atria in the porcine AF model, suggesting that active STAT3 regulates transcription during AF [110]. However, the exact role of Rac1 in mediating AngII-induced STAT3 activation in atrial remodeling and AF remains unknown. Expression of CTGF and the intercalated disc proteins N-cadherin and connexin 43 (Cx43) is also increased in the atria of AF patients and Rac1^ET^ mice [108]. In neonatal cardiomyocytes and fibroblasts, AngII treatment promotes the expression of CTGF, which leads to upregulation of N-cadherin and Cx43, while pretreatment with NSC23766 abolishes these effects [108]. Therefore, Rac1 may act as a mediator of CTGF activation to facilitate structural remodeling through N-cadherin and Cx43.

Rac1 has also been studied for its role in ventricular arrhythmias. Cardiomyocyte-specific Rac1 knockdown mice as well as mice treated with NSC23766 are protected against ventricular arrhythmia after I/R which is thought to be due to decreased Ca^2+^ release from the sarcoplasmic reticulum (SR) [113]. In contrast, Rac1^ET^ mice display increased SR Ca^2+^ leak, leading to increased diastolic calcium levels and reduced calcium transient amplitude [114]. Importantly, ROS production was lower in Rac1^ET^ ventricular myocytes, suggesting that Rac1-dependent regulation of myocyte calcium handling may occur in a NOX2-independent manner [114].

### 3.2 PAK1 in cardiac arrhythmia

The role of PAK1 in the regulation of cardiac ion channels and sarcomeric proteins has been summarized previously [84,115,116]. Loss of PAK1 results in dysregulation of protein dephosphorylation by protein phosphatase 2A (PP2A) [117,118] and disruption of ion channel activity and intracellular Ca^2+^ homeostasis [115]. For instance, in ventricular tissue from cardiomyocyte *Pak1*-deleted hearts, SR Ca^2+^-ATPase 2a (SERCA2a) is not transcriptionally upregulated in response to chronic β-adrenergic stimulation, which is associated with irregularities in calcium handling including delayed SR Ca^2+^ store recovery and Ca^2+^ waves [119]. In addition, PAK1 could play a role in the relationship between Rac1 palmitoylation and protein phosphatase activity proposed previously [24]. The importance of PAK1 for cardiomyocyte PP2A activity suggests that hyperphosphorylation of PKA substrates observed in Rac1^cKI^ hearts resistant to Rac1 palmitoylation at cysteine-178 following β-adrenergic stimulation may be a result of impaired PAK1 function or subcellular targeting of PAK1 or its effector PP2A (Fig. 2).

PAK1 is important for proper calcium handling to maintain sinus rhythm [84]. Cardiomyocyte-specific *Pak1* knockout mice develop more frequent ventricular arrhythmias in response to both acute and chronic isoproterenol administration [119] and after ischemic preconditioning, which is cardioprotective in wildtype mice [120]. Atrial arrhythmic activity also increases in mice lacking cardiomyocyte PAK1 in response to carbachol administration or atrial burst pacing [121]. Furthermore, a PAK1-activating peptide reduced ventricular tachycardia and ventricular fibrillation susceptibility in *ex vivo* mouse hearts and the occurrence of spontaneous calcium sparks and waves in ventricular myocytes treated with AngII [122].

PAK1 has emerged as a potential therapeutic target for anti-arrhythmic drugs due to its cardioprotective effects. Treatment of rat hearts *ex vivo* with FTY720 attenuated the decrease in PAK1 and Akt activity and reduced the frequency of arrhythmic events after I/R [123]. PAK1-deficient ventricular myocytes have elevated reverse-mode sodium‑calcium exchanger (NCX) activity that is abolished by treatment with a ROS scavenger [121]. This increase in NCX-mediated Ca^2+^ entry in *Pak1*-deleted myocytes causes more frequent Ca^2+^ release events induced by AngII or ischemia [121,124]. In contrast, PAK1 activation with FTY720 in a canine AF model decreased NCX activity and spontaneous Ca^2+^ release [124]. Thus, PAK1 activation with FTY720 or similar compounds may be protective for both pathologic remodeling and arrhythmia.

### 3.3 NOX2 in cardiac arrhythmia

The involvement of NOX-mediated ROS production in atrial fibrillation has been thoroughly reviewed [125]. Importantly, NOX activity increased in the left atrial appendage of pigs after AF that was associated with more active Rac1 [126]. NOX activity was also elevated in canine ventricular myocardium after induction of tachycardia that was associated with increased levels of Rac1 and p47^phox^ in SR-enriched membranes [127]. Isolated myocytes from NOX2 transgenic mice had increased cell shortening and calcium transient amplitude along with increased SR Ca^2+^ load, SERCA2a activity, and phosphorylation of phospholamban at the PKA-regulated serine-16 phospho-site [82]. Incubation of canine cardiomyocytes with NADPH leads to ryanodine receptor 2 (RyR2) S-glutathionylation and more rapid calcium release from the SR, which is reversed by treatment with the NOX inhibitor apocynin [127]. NOX inhibition also reduced the occurrence of ventricular tachycardia and fibrillation after coronary artery occlusion and lowered active Rac1 levels in the myocardium [128]. Rac1/NOX2-regulated ROS production is also enhanced following stretch, termed “X-ROS” signaling, which elicits calcium sparks in the T-tubular region, desensitizing RyR2 and evoking arrhythmogenic Ca^2+^ waves in diseased cardiomyocytes [129]. Thus, NOX2-generated ROS impacts cardiomyocyte calcium handling and propensity for arrhythmia in addition to hypertrophy.

## 4 Discussion

As highlighted above, an abundance of published data suggest important roles for Rac1 signaling in response to cardiac stress. Rac1 is required for proper cardiac development *in utero* as loss of Rac1 in embryogenesis leads to ventricular noncompaction and other structural defects [105,106,130]. Consistently, data from *in vitro* and *in vivo* studies show that Rac1 signaling is largely dispensable for cardiac homeostasis in the adult heart, as perinatal Rac1 deletion in cardiomyocytes has unremarkable effects on basal cardiac structure, function, and signaling. However, Rac1 clearly plays critical roles in cardiac stress adaptation as evidenced by several studies highlighting impaired remodeling in response to hypertrophic stimuli in mice with genetic manipulation of Rac1.

Therapeutic approaches to inhibit Rac1 signaling in cardiomyocytes have been proposed as potential treatments for heart failure. Administration of statins not only lowers cholesterol but also impairs protein prenylation through inhibition of the mevalonate pathway. Statins have been shown to reduce Rac1 activity in cardiomyocytes [5,60,61] and protect against AngII or pressure overload-induced cardiac hypertrophy *in vivo* [60,61]. Thus, part of the therapeutic benefits of statins on cardiovascular disease in humans may be in part due to inhibition of cardiomyocyte Rac1 signaling.

The compound NSC23766, a Rac1 inhibitor [131], has been used in several studies to assess the functions of Rac1 in disease settings. Indeed, NSC23766 reduces Rac1 activation in response to high glucose [42] and TAC [6]. NSC23766 does not directly inhibit Rac1 but rather disrupts the interaction between Rac1 and certain GEFs such as Trio and Tiam1 [131]. However, NSC23766 inhibits M2 muscarinic receptors in NRCMs at similar doses that have been used to inhibit Rac1 GTP-loading [132] and is proposed to inhibit NMDA receptors as well [133]. Despite these confounding factors, it is important to note that NSC23766 administration has been shown to reduce cardiac hypertrophy and improve systolic function following TAC-induced pressure overload [6], suggesting potential therapeutic benefit of NSC23766 or related compounds for heart disease.

Specific inhibitors of NADPH oxidases have been difficult to develop due to the homology between catalytic domains of different NOX enzymes [134]. Because NOX2 relies on Rac1 activation and the subsequent translocation of other auxiliary subunits (p67^phox^, p47^phox^, p40^phox^) to the plasma membrane-localized NOX2 (gp91^phox^)/p22^phox^ heterodimer, reducing NOX2 activity by interfering with the activity of its auxiliary subunits has been proposed. Indeed, an inhibitor targeting the Rac1-p67^phox^ interaction, Phox-I, was developed and shown to prevent superoxide production in neutrophils [135], however the effects of this inhibitor on cardiomyocyte Rac1 signaling and ROS production are yet to be reported. Additionally, GSK2795039 was developed as a NOX2-selective inhibitor [136] and improves systolic function and reduces cardiac hypertrophy in mouse models of myocardial infarction [137] and doxorubicin-induced cardiomyopathy [138,139], suggesting inhibition of NOX2-generated superoxide production may be a promising therapeutic approach.

FTY720, an FDA-approved sphingosine derivative, is protective in several models of cardiac hypertrophy and proposed to function through activation of cardiomyocyte PAK1 as its antihypertrophic effects are not observed in PAK1 conditional knockout mice [86]. However, it is unknown if FTY720 requires Rac1 to activate PAK1 or if it does so by circumventing Rac1. Additionally, while FTY720 treatment ameliorates pathologic cardiac remodeling in mice, the only data in human patients to date described reduced heart rate following FTY720 treatment [140] without descriptions of effects on cardiac structure or function.

One limit to targeting Rac1 signaling *in vivo* is the ubiquitous expression of Rac1 and its important physiologic roles in many cell types including other cell types in the heart, such as cardiac fibroblasts and endothelial cells. Thus, identifying cardiomyocyte-specific mechanisms that either regulate or are regulated by Rac1 signaling could aid in the identification of novel targets for therapeutic development in heart failure. For example, targeting of ASK1 with an ASK1 inhibitor, selonsertib, which prevented AngII-induced cardiac hypertrophy and fibrosis in mice [71], or activation of PAK1 as mentioned above, are potentially indirect means of effectively targeting Rac1-regulated pathogenic signaling in cardiomyocytes. Our group demonstrated that palmitoylation cycling of Rac1 at cysteine-178 is critical for cardiac stress adaptation [24] and thus, it is critical that any therapies directly targeting Rac1 avoid inhibiting palmitoylation of Rac1 to prevent sustained cardiac PKA signaling that can enhance cardiac workload and promote cardiac decompensation in response to chronic stress. Conversely, *Zdhhc3* overexpression causes severe dilated cardiomyopathy that is associated with an increase in palmitoylated Rac1 levels [22], suggesting that both enhancing or disrupting Rac1 palmitoylation at cysteine-178 can have detrimental effects on cardiac adaptation and remodeling.

While three decades of work have identified important roles of Rac1 signaling in cardiomyocytes, there remain several areas that are poorly defined. One major limitation is the lack of identification of specific GEFs and GAPs that regulate Rac1 in cardiomyocytes and the contexts in which particular GEFs/GAPs are activated. Due to the wide diversity and variable specificity of GEFs and GAPs that regulate Rac1 and other Rho family GTPases, studies aimed at identifying the primary enzymes that regulate Rac1 activity in cardiomyocytes will further enhance our understanding of Rac1 signaling in cardiomyocytes. Future studies should be directed at understanding the regulatory enzymes and spatial organization that govern Rac1 signaling in cardiomyocytes to uncover novel signaling circuitry for potential targeting in cardiac diseases.

## CRediT authorship contribution statement

**James P. Teuber:** Writing – review & editing, Writing – original draft, Conceptualization. **Rachel E. Scissors:** Writing – review & editing, Writing – original draft, Conceptualization. **Matthew J. Brody:** Writing – review & editing, Conceptualization.

## Funding sources

This work was supported by grants from the 10.13039/100000050National Heart, Lung, and Blood Institute (R01HL167778 to MJB and F31HL165180 to JPT) and the 10.13039/100000968American Heart Association (898429 to JPT).

## Declaration of competing interest

The authors declare that no competing interests exist.
